# Supervised, semi-supervised and unsupervised inference of gene regulatory networks

**DOI:** 10.1093/bib/bbt034

**Published:** 2013-05-21

**Authors:** Stefan R. Maetschke, Piyush B. Madhamshettiwar, Melissa J. Davis, Mark A. Ragan

**Keywords:** gene regulatory networks, simulation, gene expression data, machine learning

## Abstract

Inference of gene regulatory network from expression data is a challenging task. Many methods have been developed to this purpose but a comprehensive evaluation that covers unsupervised, semi-supervised and supervised methods, and provides guidelines for their practical application, is lacking.

We performed an extensive evaluation of inference methods on simulated and experimental expression data. The results reveal low prediction accuracies for unsupervised techniques with the notable exception of the Z-SCORE method on knockout data. In all other cases, the supervised approach achieved the highest accuracies and even in a semi-supervised setting with small numbers of only positive samples, outperformed the unsupervised techniques.

## INTRODUCTION

Mapping the topology of gene regulatory networks is a central problem in systems biology. The regulatory architecture controlling gene expression also controls consequent cellular behavior such as development, differentiation, homeostasis and response to stimuli, while deregulation of these networks has been implicated in oncogenesis and tumor progression [1]. Experimental methods based, for example, on chromatin immunoprecepitation, DNaseI hypersensitivity or protein-binding assays are capable of determining the nature of gene regulation in a given system, but are time-consuming, expensive and require antibodies for each transcription factor [2]. Accurate computational methods to infer gene regulatory networks, particularly methods that leverage genome-scale experimental data, are urgently required not only to supplement empirical approaches but also, if possible, to explore these data in new more-integrative ways.

Many computational methods have been developed to infer regulatory networks from gene expression data, predominately using unsupervised techniques. Several comparisons have been made of network inference methods, but a comprehensive evaluation that covers unsupervised, semi-supervised and supervised methods is lacking, and many questions remain open. Here we address fundamental questions, including which methods are suitable for what kinds of experimental data types, and how many samples these methods require. In the following, we firstly review large-scale comparisons (more than five methods), before discussing evaluations focused on supervised and semi-supervised methods. Finally we discuss the remaining smaller comparisons with an application-specific focus.

The most recent and largest comparison so far has been performed by Madhamshettiwar *et al.* [3]. They compared the prediction accuracy of eight unsupervised and one supervised method on 38 simulated data sets. The methods showed large differences in prediction accuracy but the supervised method was found to perform best, despite the parameters of the unsupervised methods having been optimized. Here we extend this study to 17 unsupervised methods and include a direct comparison with supervised and semi-supervised methods on a wide range of networks and experimental data types (knockout, knockdown and multifactorial).

Another comprehensive evaluation, limited to unsupervised methods, has been performed as part of the Dialogue for Reverse Engineering Assessments and Methods (DREAM), an annual open competition in network inference [4–8]. Results from DREAM highlight that network inference is a challenging problem. To quote Prill *et al.* [7], ‘The vast majority of the teams’ predictions were statistically equivalent to random guesses’. However, an important result of the DREAM competition is that under certain conditions, simple methods can perform well: ‘ … the z-score prediction would have placed second, first, and first (tie) in the 10-node, 50-node, and 100-node subchallenges, respectively’ [7].

Unsupervised methods rely on expression data only but tend to achieve lower prediction accuracies than supervised methods [3, 9, 10]. By contrast, supervised methods require information about known interactions for training, and this information is typically sparse. Semi-supervised methods reflect a compromise and can be trained with much fewer interaction data, but usually are not as accurate predictors as supervised methods. One of the few comparisons with supervised methods was performed by Mordelet and Vert [9]. They evaluated supervised inference of regulatory networks (SIRENE) in comparison to context likelihood of relatedness (CLR), algorithm for the reconstruction of accurate cellular networks (ARACNE), relevance networks (RN) and a Bayesian network (BN) on an *Escherichia coli* benchmark data set by Faith *et al.* [11] and found that the supervised method considerably outperformed the unsupervised techniques.

Cerulo *et al.* [10] compared supervised and semi-supervised support vector machines (SVMs) with two unsupervised methods and found the former superior. Our evaluation uses similar supervised and semi-supervised methods but includes many more unsupervised methods, distinguishes between experimental types and performs repeats, resulting in a more complete picture. A related evaluation by Schaffter *et al.* [12] compared six unsupervised methods on larger networks with 100, 200 and 500 nodes and simulated expression data. Again the z-score method was found to be one of the top performers in knockout experiments.

Several smaller evaluations have been performed but are largely restricted to four unsupervised methods (ARACNE, CLR, MRNET and RN) in comparisons with a novel approach on small data sets. The ARACNE method was introduced by Margolin *et al.* [13] and showed superior precision and recall when compared with RN and a BN algorithm on simulated networks. Meyer *et al.* [14] compared all four unsupervised inference algorithms on large yeast subnetworks (100 up to 1000 nodes) using simulated expression data, and Altay and Emmert-Streib [15] investigated the bias in the predictions of those algorithms. Faith *et al.* [11] evaluated CLR, ARACNE, RN and a linear regression model on *E. coli* interaction data from RegulonDB and found CLR to outperform the other methods. Lopes *et al.* [16] studied the prediction accuracy of ARACNE, MRNET, CLR and SFFS + MCE, a feature selection algorithm, on simulated networks and found the latter superior for networks with small node degree. Haynes and Brent [17] developed a synthetic regulatory network generator (GRENDEL) and measured the prediction accuracy of ARACNE, CLR, DBmcmc and Symmetric-N for various network sizes and different experimental types. Werhli *et al.* [18] compared RN, graphical Gaussian models (GGMs) and BNs on the Raf pathway, a small cellular signaling network with 11 proteins, and on simulated data. BNs and GGMs were found to outperform RN on observational data. Camacho *et al.* [19] compared regulatory strengths analysis, reverse engineering by multiple regression, partial correlations (PC) and dynamic BNs on a small simulated network with 10 genes, with different levels of noise. In the noise-free scenario, the PC method showed the highest accuracy. Finally, Cantone *et al.* [20] constructed a small, synthetic, *in vivo* network of five genes and measured time series and steady-state expression. In an evaluation of BANJO, ARACNE and two models based on ordinary differential equations, they found the latter two to achieve the highest accuracies. Bansal *et al.* [21] also evaluated BANJO, ARACNE and ordinary differential equations but on random networks and simulated expression data.

In the following sections, we first describe the different inference methods in detail, before evaluating their prediction accuracies on simulated and experimental gene expression data and regulatory networks of varying size. We continue with a discussion of the prediction results and conclude with guidelines for the use of the evaluated methods.

## METHODS

We compared the prediction accuracy of unsupervised, semi-supervised and supervised network inference methods. Unsupervised methods do not use any data to adjust internal parameters. Supervised methods, on the other hand, exploit all given data to optimize parameters such as weights or thresholds. Semi-supervised methods use only part of the data for parameter optimization, for instance, a subset of known network interactions. Note that unsupervised methods would be rendered (at least) semi-supervised by optimizing their parameters on network data.

The inference methods we evaluate here aim to recreate the topology of a genetic regulatory network—that is, a network of gene-to-gene physical regulatory interactions, some of which, however, might be hidden by shortcuts [8]—based on expression data only. In this context, the accuracy of a method is assessed by the extent to which the network it infers is similar to the true regulatory network. Because many methods are not designed to infer self-interactions or interaction direction we disregard directed edges and self-interactions. Following other [9, 17, 22,] we quantify similarity by the Area under the Receiver Operator Characteristic curve (AUC)
(1)
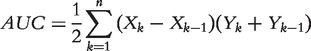

where *X_k_* is the false-positive rate and *Y_k_* is the true-positive rate for the *k*-th output in the ranked list of predicted edge weights. An AUC of 1.0 indicates perfect prediction, while an AUC of 0.5 indicates a performance no better than random predictions.

Note that in contrast to other measures such as F1 score, Matthews correlation, recall or precision [23], AUC does not require choice of a threshold to infer interactions from predicted weights; rather, it compares the predicted weights directly to the topology of the true network. In the Supplementary Material, we nonetheless report results based on F1 score and Matthews correlation.

To avoid discrepancies between the gene expression values generated by true regulatory networks and the actually known, partial networks, we performed evaluations on simulated, steady-state expression data, generated from subnetworks extracted from *E. coli* and *Saccharomyces cerevisiae* networks. This allowed us to assess the accuracy of an algorithm against a perfectly known true network [21]. We used *GeneNetWeaver* [12, 24] and *SynTReN* [25] to extract subnetworks and to simulate gene expression data.

*GeneNetWeaver* has been part of several evaluations, most prominently the DREAM challenges. The simulator extracts subnetworks from known interaction networks such as those of *E. coli* and *S. cerevisiae*, emulates transcription and translation, and uses a set of ordinary differential equations describing chemical kinetics to generate expression data for knockout, knockdown and multifactorial experiments.

To simulate knockout experiments, the expression value of each gene is in turn set to zero, whereas for knockdown experiments, the expression value is halved. In multifactorial experiments, the expression levels of a small number of genes are perturbed by a small random amount. In contrast to the DREAM challenge, we do not provide to the inference algorithms metadata such as which gene has been knocked out or knocked down. All unsupervised methods see only expression data, while supervised methods see expression data plus interaction data.

*SynTReN* is a similar but older simulator. Subgraphs are also extracted from *E. coli* and *S. cerevisiae* networks but it simulates only the transcription level and multifactorial experiments. However, *SynTReN* is faster than *GeneNetWeaver* and allows one to vary the sample number independently of the network size.

To enable a comprehensive and fair comparison, we evaluated the prediction accuracies of these inference methods on subnetworks with different numbers of nodes (10, … ,110) extracted from *E. coli* and *S. cerevisiae*, and used three experimental data types [(knockout, knockdown, multifactorial) with varying sample set sizes (10, … ,110)] simulated by GeneNetWeaver and SynTReN.

We performed no parameter optimization for unsupervised methods because this would require training data (known interactions) and render those methods supervised. For the supervised and semi-supervised methods, 5-fold cross-validation was applied and parameters were optimized on the training data only.

In addition to simulated data, we also evaluated all methods on two experimental data sets originating from the fifth DREAM systems biology challenge [8]. Specifically, we downloaded an *E. coli* network with 296 regulators, 4297 genes and the corresponding expression data with 487 samples, and an *S. cerevisiae* network with 183 regulators, 5667 genes and expression data with 321 samples. Both data sets are described in detail in the Supplementary Material of [8]. The following sections describe the inference methods in detail.

### Unsupervised

This section describes the evaluated unsupervised methods. CLR, ARACNE, MRNET and MRNET-B are part of the R package ‘minet’ and were called with their default parameters [26], with the exception of ARACNE. With the default parameter 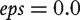, ARACNE performed poorly and we used 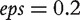 instead. Similarly, gene network inference with ensemble of trees (GENIE) [27], MINE [28] and partial correlation and information theory (PCIT) [29] were installed and evaluated with default parameters. All other methods were implemented according to their respective publications. SPEARMAN-C, EUCLID and SIGMOID are implementations of our own inference algorithms.

#### Correlation

Correlation-based network inference methods assume that correlated expression levels between two genes are indicative of a regulatory interaction. Correlation coefficients range from +1 to −1 and a positive correlation coefficient indicates an activating interaction, while a negative coefficient indicates an inhibitory interaction. The common correlation measure by Pearson is defined as
(2)
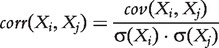

where *X_i_* and *X_j_* are the expression levels of genes *i* and *j*, 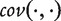 denotes the covariance, and 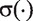 is the standard deviation. Pearson’s correlation measure assumes normally distributed values, an assumption that does not necessarily hold for gene expression data. Therefore rank-based measures are frequently used, with the measures by Spearman and Kendall being the most common. Spearman’s method is simply Pearson’s correlation coefficient for the ranked expression values, and Kendall’s τ coefficient is computed as
(3)
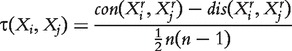

where 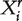 and 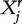 are the ranked expression profiles of genes *i* and *j*. 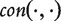 denotes the number of concordant and 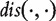 the number of disconcordant value pairs in 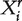 and 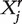, with both profiles being of length *n*.

Because our evaluation of prediction accuracy does not distinguish between inhibiting and activating interactions, the predicted interaction weights are computed as the absolute value of the correlation coefficients
(4)
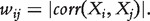



#### SPEARMAN-C

SPEARMAN-C is a modification of Spearman’s correlation coefficient where we attempted to favor hub nodes, which have many, strong interactions. The correlation coefficient is corrected by multiplying it by the mean correlation of gene *i* with all other genes *k*, and the absolute value is taken as the interaction weight
(5)
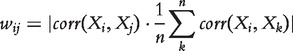

where 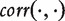 is Spearman’s correlation coefficient.

#### Weighted gene co-expression network analysis

WGCNA [30] is a modification of correlation-based inference methods that amplifies high correlation coefficients by raising the absolute value to the power of β (‘softpower’).
(6)
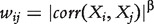

with 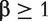. Because softpower is a nonlinear but monotonic transformation of the correlation coefficient, the prediction accuracy measured by AUC will be no different from that of the underlying correlation method itself. Consequently we show only results for correlation methods but not for the WGCNA modification, which would be identical.

#### Relevance networks

RN by Butte and Kohane [31] measure the mutual information (MI) between gene expression profiles to infer interactions. The MI *I* between discrete variables *X_i_* and *X_j_* is defined as
(7)
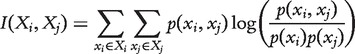

where 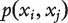 is the joint probability distribution of *X_i_* and *X_j_*, and 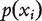 and 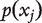 are the marginal probabilities. *X_i_* and *X_j_* are required to be discrete variables. We used equal-width binning for discretization and empirical entropy estimation as described by Meyer *et al.* [26].

#### Context likelihood of relatedness

CLR [11] extends the RN by taking the background distribution of the MI values 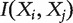 into account. The most probable interactions are those that deviate most from the background distribution, and for each gene *i*, a maximum z-score *z_i_* is calculated as
(8)
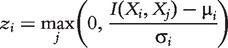

where 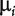 and 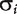 are the mean value and standard deviation, respectively, of the MI values 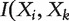), 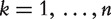. The interaction *w_ij_* between two genes *i* and *j* is then defined as
(9)
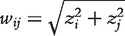

The background correction step aims to reduce the prediction of false interactions based on spurious correlations and indirect interactions.

#### Algorithm for the reconstruction of accurate cellular networks

ARACNE [13] is another modification of the RN that applies the Data Processing Inequality (DPI) to filter out indirect interactions. The DPI states that, if gene *i* interacts with gene *j* via gene *k*, then the following inequality holds:
(10)
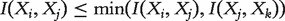

ARACNE considers all possible triplets of genes (interaction triangles) and computes the MI values for each gene pair within the triplet. Interactions within an interaction triangle are assumed to be indirect and are therefore pruned if they violate the DPI beyond a specified tolerance threshold *eps*. We used a threshold of 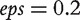 for our evaluations.

#### Partial correlation and information theory

PCIT [29] is similar to ARACNE. PCIT extracts all possible interaction triangles and applies the DPI to filter indirect interactions, but uses partial correlation coefficients instead of MI as interaction weights. The partial correlation coefficient 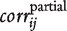 between two genes *i* and *j* within an interaction triangle 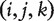 is defined as
(11)
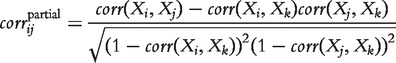

where 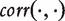 is Person’s correlation coefficient. The partial correlation coefficient aims to eliminate the effect of the third gene *k* on the correlation of genes *i* and *j*.

#### MRNET

MRNET [14] uses the MI between expression profiles and a feature selection algorithm [minimum-redundancy–maximum-relevance (MRMR)] to infer interactions between genes. More precisely, the method places each gene in the role of a target gene *j* with all other genes *V* as its regulators. The MI between the target gene and the regulators is calculated and the MRMR method is applied to select the best subset of regulators. MRMR step-by-step builds a set *S* by selecting the genes *i^MRMR^* with the largest MI value and the smallest redundancy based on the following definition:
(12)
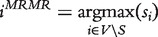

with 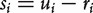. The relevance term 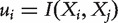 is thereby the MI between gene *i* and target *j*, and the redundancy term *r_i_* is defined as
(13)
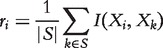

Interaction weights *w_ij_* are finally computed as 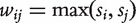.

#### MRNET-B

MRNET-B is a modification of MRNET that replaces the forward selection strategy to identify the best subset of regulator genes by a backward selection strategy followed by a sequential replacement [32].

#### Gene network inference with ensemble of trees

GENIE is similar to MRNET in that it also lets each gene take on the role of a target regulated by the remaining genes and then uses a feature selection procedure to identify the best subset of regulator genes. In contrast to MRNET, Random Forests and Extra-Trees are used for regression and feature selection [27] rather than MI and MRMR.

#### SIGMOID

SIGMOID models the regulation of a gene by a linear combination with soft thresholding. The predicted expression value 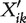 of gene *i* at time point *k* is described by the sum over the weighted expression values *X_jk_* of the remaining genes, constrained by a sigmoid function 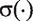.
(14)
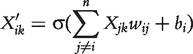

(15)
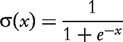

The regulatory weights *w_ij_* are determined by minimizing the following quadratic error function over the predicted expression values 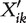 and the observed values *X_ik_*:
(16)
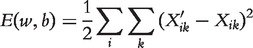

Finally, the interaction weights 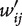 for the undirected network are computed by averaging over the forward and backward weights:
(17)
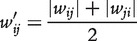



#### Mass–distance

MD by Yona *et al.* [33] is a similarity measure for expression profiles. It estimates the probability to observe a profile inside the volume delimited by the profiles. The smaller the volume, the more similar are the two profiles. Given two expression profiles *X_i_* and *X_j_*, the total probability mass of samples whose *k*-th feature is bounded between the expression values *X_ik_* and *X_jk_* is calculated as
(18)
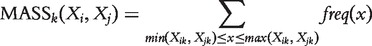

where *freq*(*x*) is the empirical frequency. The mass distance 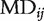 is defined as the total volume of profiles bounded between the two expression profiles *X_i_* and *X_j_* and is estimated by the product over all coordinates *k*.
(19)
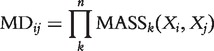

where *n* is the length of the expression profiles. Because the 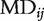 is symmetric and positive, we interpret it directly as an interaction weight *w_ij_*.

#### Mutual rank

MR by Obayashi and Kinoshita [34] uses ranked Pearson’s correlation as a measure to describe gene co-expression. For a gene *i*, first Pearson’s correlation with all other genes *k* is computed and ranked. Then the rank achieved for gene *j* is taken as score to describe the similarity of the gene expression profiles *X_i_* and *X_j_*:
(20)
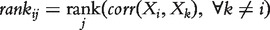

with 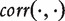 being Pearson’s correlation coefficient. The final interaction weight *w_ij_* is calculated as the geometric average of the ranked correlation between gene *i* and *j* and vice versa:
(21)
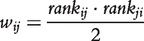



#### Maximal information nonparametric exploration

MINE is a class statistics by Reshef [28]. The maximal information coefficient (MIC) is part of this class and a novel measure to quantify nonlinear relationships. We computed the 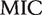 for expression profiles *X_i_* and *X_j_* and interpreted the 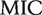 score as an interaction weight
(22)
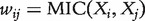



#### EUCLID

EUCLID is a simple method that uses the euclidean distance between the normalized expression profiles 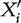 and  of two genes as interaction weights
(23)
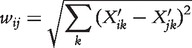

where profiles are normalized by computing the absolute difference of expression values *X_ik_* to the median expression in profile *X_i_*
(24)
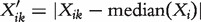



#### Z-SCORE

Z-SCORE is a network inference strategy by Prill *et al.* [7] that takes advantage of knockout data. It assumes that a knockout affects directly interacting genes more strongly than others. The z-score *z_ij_* describes the effect of a knockout of gene *i* in the *k*-th experiment on gene *j* as the normalized deviation of the expression level *X_jk_* of gene *j* for experiment *k* from the average expression 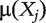 of gene *j*:
(25)
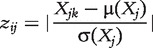

The original Z-SCORE method requires knowledge of the knockout experiment *k* and is therefore not directly applicable to data from multifactorial experiments. The method, however, can easily be generalized by assuming that the minimum expression value within a profile indicates the knockout experiment [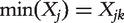]. Equation (25) then becomes
(26)
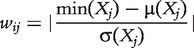

and the method can be applied to knockout, knockdown and multifactorial data. Note that *z_ij_* is an asymmetric score and we therefore take the maximum of *z_ij_* and *z_ji_* to compute the final interaction weight *w_ij_* as
(27)
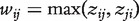



### Supervised

A great variety of different supervised machine learning methods has been developed. We limit our evaluation to SVMs because they have been successfully applied to the inference of gene regulatory networks [9] and can easily be trained in a semi-supervised setting [10]. We used the SVM implementation *SVMLight* by Joachims [35] for all evaluations.

SVMs are trained by maximizing a constrained, quadratic optimization problem over Lagrange multipliers α:
(28)
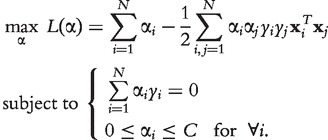

The labels *y_i_* determine the class to which feature vector 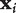 belongs and *C* is the so-called *complexity* parameter that needs to be tuned for optimal prediction performance. Once the optimal Lagrange multipliers α are found, a feature vector can be classified by its signed distance 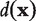 to the decision boundary, which is computed as
(29)
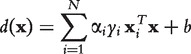

The distance 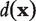 can be interpreted as a confidence value. The larger the absolute distance, the more confident the prediction, and similar to a correlation value we interpret the distance as an interaction weight.

In contrast to unsupervised methods, e.g. correlation methods, the supervised approach does not directly operate on pairs of expression profiles but on feature vectors that can be constructed in various ways. We computed the outer product of two gene expression profiles *X_i_* and *X_j_* to construct feature vectors:
(30)
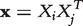

The outer product was chosen because it is commutative, and predicted interactions are therefore symmetric and undirected. A sample set for the training of the SVM is then composed of feature vectors 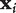 that are labeled 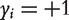 for gene pairs that interact and 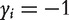 for those that do not interact.

**Figure 1:**
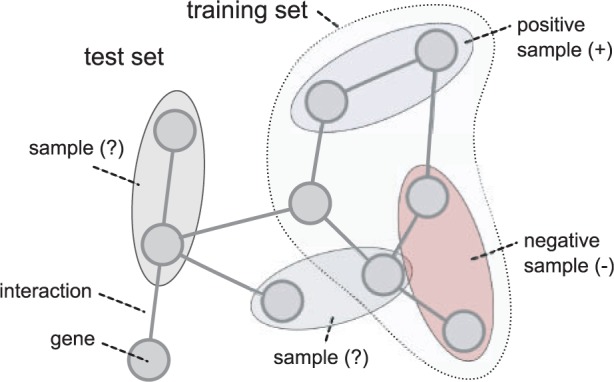
Extraction of samples for the training and test set from a gene interaction network.

If all gene pairs are labeled, all network interactions would be known and prediction would be unnecessary. In practice and for evaluation purposes, training is therefore performed on a set of labeled samples, and predictions are generated for the samples of a test set. Figure 1 depicts the concept. All samples within the training set are labeled and all remaining gene pairs serve as test samples.

Note that the term ‘sample’ in the context of supervised learning refers to a feature vector derived from a pair of genes and their expression profiles, whereas a sample in an expression data set refers to the gene expression values for a single experiment, e.g. a gene knockout.

We evaluate the prediction accuracy of the supervised method by generating labeled feature vectors for all gene pairs (samples) of a network. This entire sample set is then divided in to five parts. Each of the parts is used as a test set and the remaining four parts serve as a training set. The total prediction accuracy is averaged over the prediction accuracies achieved during the five iterations (5-fold cross-validation).

### Semi-supervised

Data describing regulatory networks are sparse and typically only a small fraction of the true interactions is known. The situation is even worse for negative data (non-interactions) because experimental validation largely aims to detect but not exclude interactions. The case that all samples within a training data set can be labeled as positive or negative is therefore rarely given for practical network inference problems, and supervised methods are limited to small training data sets, which negatively affects their performance.

**Figure 2:**
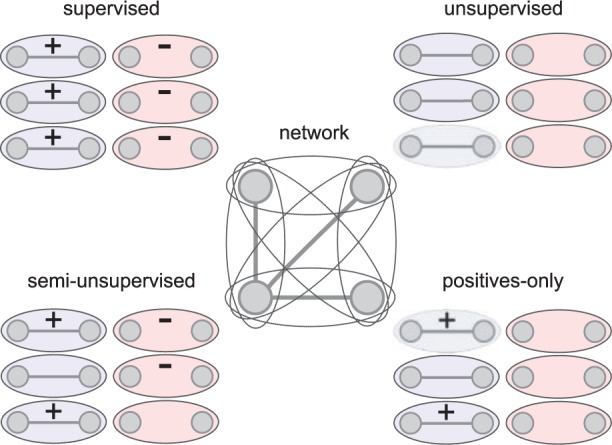
Original labeling of samples for supervised, unsupervised, semi-supervised and positives-only prediction methods. All the six samples within a sample set are generated by a four-node network with three interactions.

Semi-supervised methods strive to take advantage of the unlabeled samples within a training set by taking the distribution of unlabeled samples into account, and can even be trained on positively labeled data only. Figure 2 shows the required labeling of data for the different approaches. Supervised methods require all samples within the training set to be labeled, while unsupervised methods require no labeling at all. Semi-supervised approaches can be distinguished into methods that need positive and negative samples and methods that operate on positive samples only.

The semi-supervised method used in this evaluation is based on the supervised SVM approach described above. The only difference is in the labeling of the training set. In the semi-supervised setting, only a portion of the training samples is labeled. To enable the SVM training, which requires all samples to be labeled, all unlabeled samples within the semi-supervised training data are relabeled as negatives [10]. This approach enables a direct comparison of the same prediction algorithm trained with fully or partially labeled data.

We assigned different percentages (10%, … ,100%) of true positive and negative or positive-only labels to the training set. The prediction performance of the different approaches was then evaluated by 5-fold cross-validation, with equal training/test set sizes for the supervised, semi-supervised, positives-only and unsupervised methods compared.

## RESULTS

In the following, we first evaluate the prediction accuracy of unsupervised methods before comparing two selected unsupervised methods with supervised and semi-supervised approaches on simulated data. The last section compares unsupervised and supervised methods on experimental data.

### Unsupervised methods

**Figure 3:**
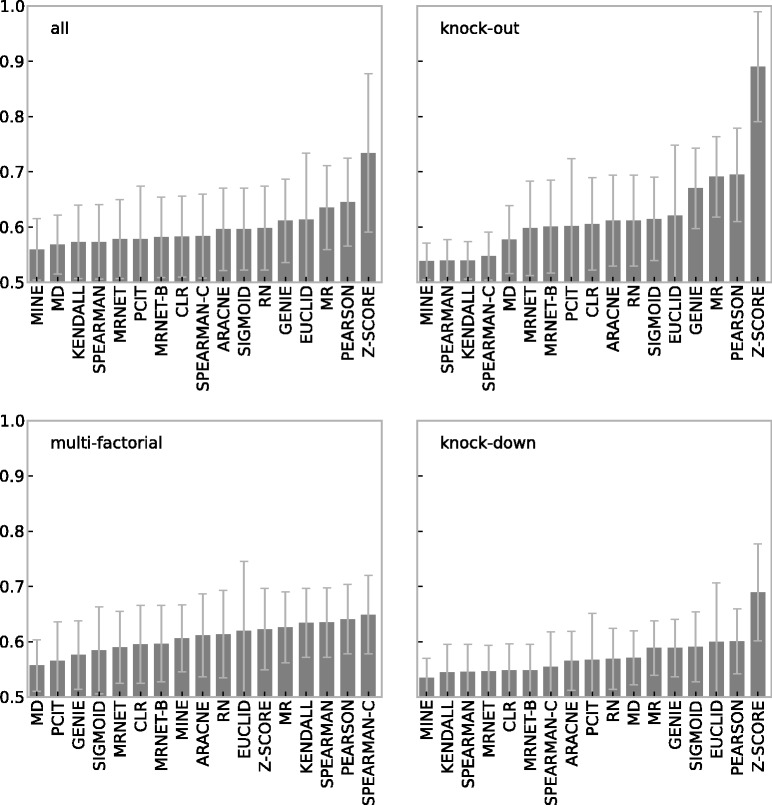
Prediction accuracy (AUC) of unsupervised methods on multifactorial, knockout, knockdown and averaged (all) data generated by GeneNetWeaver. Ten repeats over networks with 10,...,110 nodes, extracted from *E. coli* and *S. cerevisiae*. Error bars show standard deviation.

Figure 3 shows the prediction accuracies measured by AUC for all unsupervised methods for three different experimental types (knockout, knockdown and multifactorial) and the average AUC (all) over the three types. Networks with 10, 30, 50, 70, 90 and 110 nodes were extracted from *E. coli* and *S. cerevisiae* and expression data were simulated with GeneNetWeaver, with the number of samples (experiments) equal to the nodes of the network. Every evaluation was repeated 10 times, so each bar therefore represents an AUC averaged over 60 networks or 180 networks (all).

Most obvious are the large standard deviations in prediction accuracy across all methods and experimental types. For small networks, the accuracy of a method can easily vary between no better than guessing to close to perfect (see Supplementary Material). While most differences between methods are statistically significant (*P*-values <0.01 for Wilcoxon rank sum test with Bonferroni correction), differences are largely small and the ranking for most methods is therefore not stable and depends on the experimental data type, the source network, the subnetwork size and other factors (see Supplementary Material). However, a simple Pearson’s correlation is consistently the second-best performer for all experimental types.

Interestingly, rank-based correlation methods (SPEARMAN, KENDALL) that are similar to Pearson correlation perform poorly on knockout and knockdown data but well for multifactorial experiments. Obviously, a seemingly minor change from a linear correlation (Pearson) to a rank-based correlation (Spearman) has a dramatic impact on the prediction performance in the case of knockout (and knockdown) data.

With the exception of the Z-SCORE method prediction, accuracies are low in general. Z-SCORE was specifically designed for knockout data and indeed clearly outperforms all other methods for this experimental type, despite its simplicity. It is the only unsupervised method that achieves a good prediction accuracy (AUC 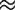 0.9).

### Network size

**Figure 4:**
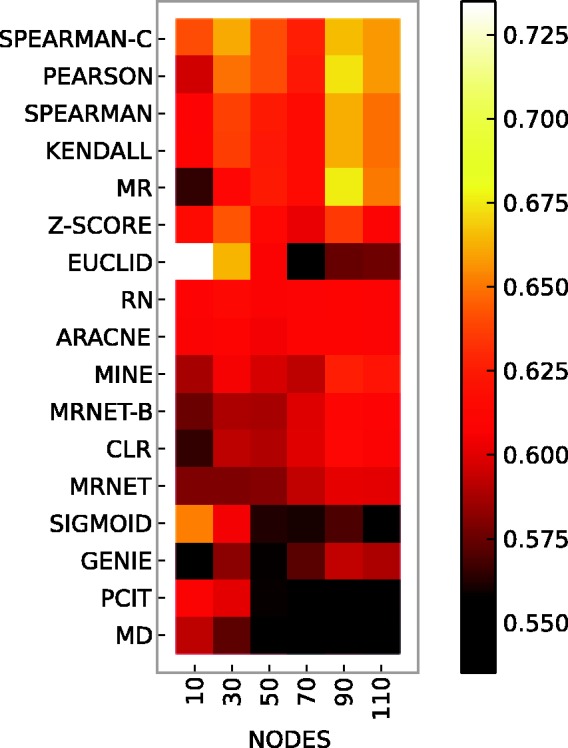
Prediction accuracy (AUC) of unsupervised methods on multifactorial data for different network sizes (nodes). Data generated by GeneNetWeaver and extracted from *E. coli* and *S. cerevisiae*.

Figure 3 summarizes results averaged over networks. We also examined how the network size impacts the prediction performance of the various methods. The heat map in Figure 4 is based on the same data as Figure 3, but shows the prediction accuracies (AUC) of the inference methods on multifactorial data for networks with different numbers of nodes (see Supplementary Material for the related figures on knockout and knockdown data).

The rows in Figure 4 are ordered according to mean AUC and the ranking is therefore identical to that in the multifactorial bar graph in Figure 3. Top performers on average are the correlation methods by Pearson, Spearman and Kendall, with the corrected Spearman method (SPEARMAN-C) achieving the highest mean AUC. However, when focusing on networks of specific size, the best performance is achieved by the EUCLID method for small networks with 10 nodes. Other methods also show different behaviors with respect to network size. Correlation methods clearly achieve higher AUCs for large networks. Similar trends can be observed for MR, MINE, GENIE, MRNET, MRNET-B and CLR. In contrast, SIGMOID, PCIT and MD decrease in prediction accuracy for growing network sizes, while the performance of RN and ARACNE is seemingly unaffected by network size within the investigated size range.

### Sample number

**Figure 5:**
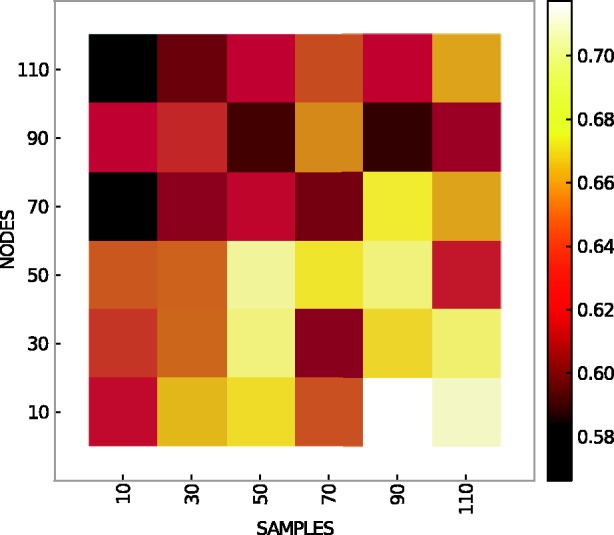
Prediction accuracy (AUC) averaged over all unsupervised methods on multifactorial for different network sizes (nodes) and sample numbers. Data generated by SynTReN and extracted from *E. coli*. Ten repeats.

Apart from the size of the network, we also expected the number of samples to have an effect on the prediction accuracy of the inference algorithms. GeneNetWeaver generates gene expression profiles with the same number of samples as network nodes (genes). We therefore used SynTReN to vary network size and sample number independently. The heat map in Figure 5 shows prediction accuracy (AUC) averaged over all inference methods for different network sizes and sample numbers. SynTReN simulates expression data for multifactorial experiments only, and networks were extracted from *E. coli*. All experiments were repeated 10 times. The results show the expected trend of improving accuracy with increasing number of samples and decreasing size of network.

However, the absolute improvements in prediction accuracy are rather small with additional data, most likely because unsupervised methods can infer only simple network topologies reliably and small sample sets are sufficient for this purpose. For instance, networks with 50 nodes are predicted with an AUC of roughly 0.65, when 50 samples are available. Increasing the sample set size to 110 raises the prediction accuracy only to an AUC of around 0.67.

### Supervised methods

Finally, we wanted to compare unsupervised with supervised and semi-supervised approaches. Because of the time-consuming training required for supervised methods, we limited our evaluation to networks with 30 nodes extracted from *E. coli* networks. Expression profiles were generated with GeneNet-Weaver, and each experiment was repeated 10 times.

**Figure 6:**
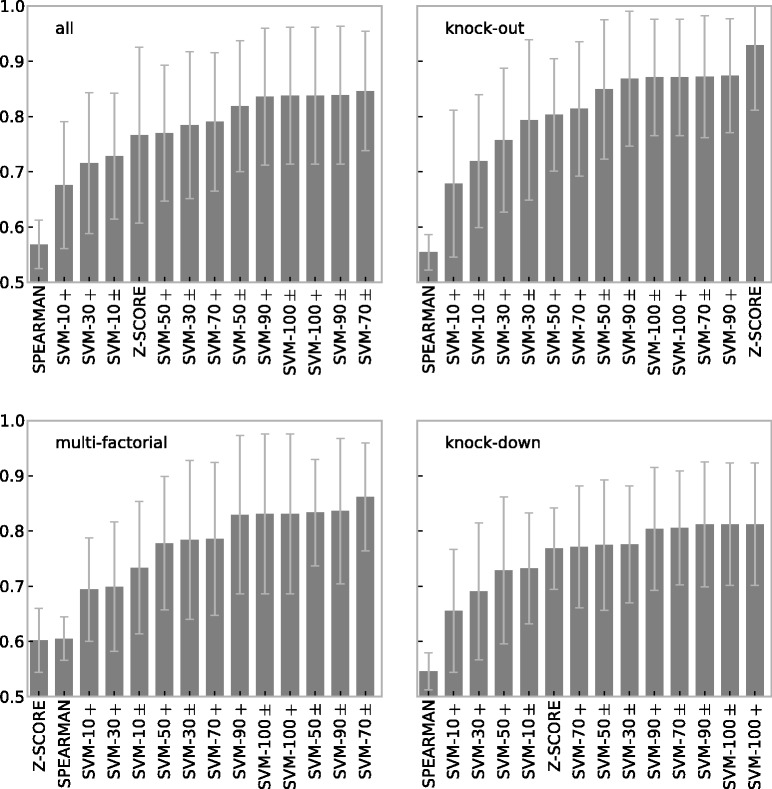
Prediction accuracy (AUC) of supervised methods on multifactorial, knockout, knockdown and averaged (all) data generated by GeneNetWeaver. Results are for 5-fold cross-validation and 10 repeats over networks with 30 nodes, extracted from *E. coli*. Error bars show standard deviation.

Figure 6 shows the prediction accuracies (AUC) for supervised and semi-supervised methods for three different experimental types (knockout, knockdown and multifactorial) and the average AUC (all) data. For direct comparison, we included two unsupervised methods (Z-SCORE, SPEARMAN) in our evaluation of supervised methods. Supervised and semi-supervised methods are labeled ‘SVM’ followed by the percentage of labeled data (10, 30, 50, 70, 90, 100%). The suffix ‘
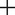
’ indicates that only positive data were used and ‘±’ indicates that positive and negative data were used. For instance, ‘SVM-70±’ describes an SVM trained on 70% of labeled data (positive and negative). All evaluations are 5-fold cross-validated and the complexity parameter *C* of the SVM was optimized via grid search (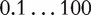) for each training fold.

The results show good prediction accuracies for supervised methods on all experimental types, with a slight advantage for knockout data. As expected, performance increases with the percentage of data labeled but there is little difference between labeling only positive data, and both positive and negative data. Apparently, supervised methods can be trained effectively even when only a portion of network interactions (positives) is known.

Even with as little as 10% of known interactions, semi-supervised methods still outperform unsupervised methods for multifactorial data. The Z-SCORE method is still the top-performing method on knockout data, but supervised methods are not far behind and considerably outperform Spearman correlation. For knockdown data, the Z-SCORE method loses its top rank, and semi-supervised methods perform better when at least 70% of the data are labeled.

To summarize, apart from the Z-SCORE method on knockout data, supervised and semi-supervised approaches considerably outperform unsupervised methods and achieve good prediction accuracies in general for networks of this size.

### Experimental data

All results in the previous sections are based on simulated data. In this section, we analyze the performance of the inference methods on the experimental data described in Methods. For more-precise comparison, we applied the same methodology and used GeneNetWeaver to randomly extract subnetworks with 30 nodes from the experimental networks. Note, however, that GeneNetWeaver was not used to simulate gene expression—here we use empirical data [8] instead—and that the *E. coli* and *S. cerevisiae* source networks are different from those on which the simulation was based.

**Figure 7:**
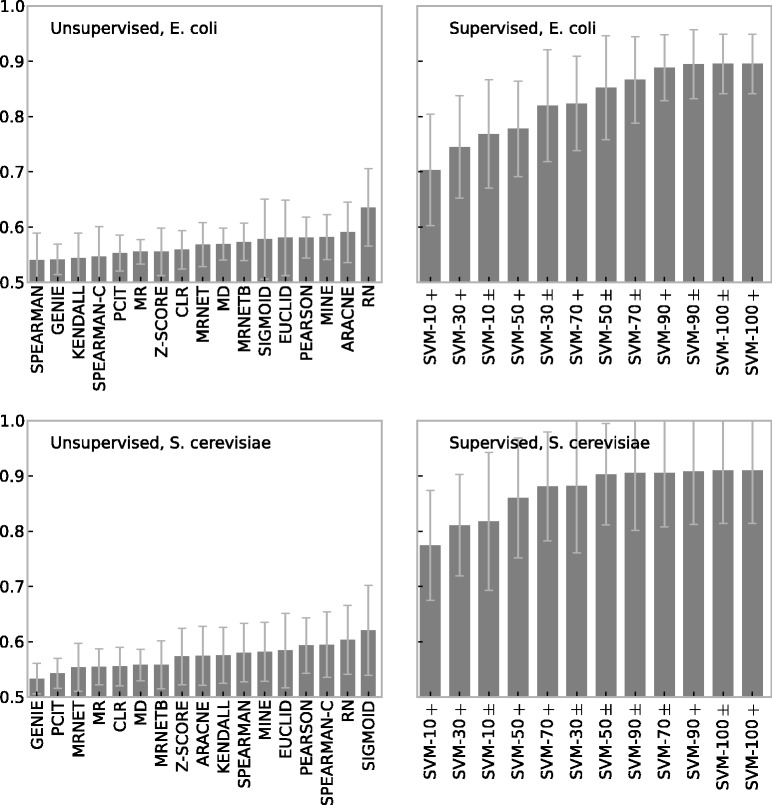
Prediction accuracy (AUC) of supervised and unsupervised methods for networks with 30 nodes extracted from *E. coli* and *S. cerevisiae*. The first 30 samples of the corresponding experimental expression data are used. AUCs are averaged over 10 repeats and error bars show standard deviation. Results for supervised methods are 5-fold cross-validated.

Figure 7 shows the prediction accuracy of the supervised and unsupervised methods for experimental networks and gene expression data of *E. coli* and *S. cerevisiae*. For better comparison, only the first 30 samples from the expression data sets were used. Results based on the complete expression data can be found in the Supplementary Material.

The results on the experimental data are in good agreement with the simulation results (Figures 3 and 6). The accuracy of the unsupervised methods remains low, while the supervised methods perform dramatically better. Even with only 10% of known interactions as a training source, the supervised methods outperform the unsupervised methods by a wide margin. The ranking of the unsupervised methods is inconsistent between data sets, but this is of little significance owing to their low accuracy and the large variances. Using the full expression data set (instead of only 30 samples) does not improve the accuracy of the best-performing unsupervised methods (see Supplementary Material).

## DISCUSSION

### Directed interactions and self-interactions

Large-scale evaluations, including DREAM, measure prediction accuracy by comparing the topology of the inferred network with a known, true network. While this true network can contain directed interactions and loops, only a small subset of methods can infer direction or loops natively (e.g. GENIE [37]). GeneNetWeaver extracts directed edges and self-interactions if present, but to make our comparison direct and fair, we subsequently ignore them in computing AUC.

### Unsupervised methods

Unsupervised methods are attractive because they do not require knowledge about the network to be inferred, and can be applied directly without a time-consuming training process. These are important practical advantages in comparison with supervised methods because reliable network data are often unavailable and training times for larger networks can become prohibitive. Parameter optimization would have removed these benefits and rendered unsupervised methods (semi-)supervised. On the other hand, as Madhamshettiwar *et al.* [3] have shown, parameter optimization can improve prediction accuracy.

### Simulated data

While simulators such as GeneNetWeaver generate expression data that are in good agreement with biological measurements [6], they remain incomplete models, e.g. posttranscriptional regulation and chromatin states are missing, and an evaluation of inference methods on real data would clearly be preferable. However, currently known network structures, even for well-characterized organisms, are fragmentary and only partially correct representations of the interactions between genes [4]. Consequently, there is an unknown but probably large discrepancy between the expression data measured and the observed part of the actual network that generates them, rendering assessment of inference methods on observed gene regulatory networks and their expression values difficult. We therefore have largely focused our evaluation on *in silico* benchmarks, but methods that fail for simulated data are unlikely to succeed in the inference of real biological networks [21].

### Linear SVMs

Another limitation of our study is the focus on linear SVMs for the evaluation of supervised and semi-supervised methods. We preferred linear SVMs over more-powerful nonlinear methods for two reasons. Firstly, linear SVMs are considerably faster to train and have fewer parameters to optimize than nonlinear SVMs—a significant advantage in a comprehensive study. Secondly, identifying a complex system with many variables (interaction weights) from a small number of samples calls for a simple predictor. We also tried to evaluate transductive SVMs [37] but found them time-consuming to train, and they achieved accuracies considerably lower than the semi-supervised SVMs (data not shown). We therefore did not perform a full evaluation and do not report results for transductive SVMs.

### Feature vectors

We construct feature vectors by computing the outer product of the expression profiles of two genes. Cerulo *et al.* [10] constructed feature vectors by concatenating the two expression profiles. The outer product results in larger feature vectors (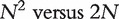) but is independent of the order of the gene pair. The training set is therefore half the size compared with the concatenation approach [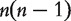] and we achieved higher prediction accuracies with the linear SVM. Cerulo *et al.* [10], however, used nonlinear SVMs (RBF) that might achieve the same or better accuracies on concatenated feature vectors but are more time-consuming to train and require two parameters (*C*, γ) to be optimized. It therefore remains an open question, which method is preferable.

SIRENE by Mordelet and Vert [9] takes a different approach, with SVMs trained on feature vectors derived from single profiles. However, it requires knowledge about the transcription factors amongst the genes, and cannot predict interactions between target genes. Because each transcription factor is assigned a separate SVM, feature vectors are of length *N* and the training set has only *n* samples, the individual SVMs can be trained efficiently, but training time is multiplied by the number of transcription factors.

### Unbalanced data sets

Gene regulatory networks tend to be sparse, with the number of positive samples (interactions) typically much smaller than the number of negative samples (non-interactions). Consequently data sets for the training of supervised methods are heavily unbalanced, and this could have a negative impact on the prediction accuracy of the classifier. We therefore tried to weight positive and negative samples inversely to their ratio, but did not observe any improvements in prediction accuracy (data not shown). All evaluations in this article were therefore performed with equally weighted (*w* = 1) samples.

### Effect of sample number

We studied the effect of sample number on the prediction accuracy using an over-determined system (more samples than genes), and found only a marginal improvement for larger sample numbers. For an under-determined system (fewer samples than genes), increasing the number of samples is likely to be more beneficial. However, large networks cannot be inferred reliably (partly owing to a lack of data) and we therefore focused our evaluation on small networks, for which sufficient data (number of samples matching the number of genes) are often available in practice.

### Network inference

The evaluation results reveal large variations in prediction accuracies across all methods. Nonlinear methods such as MINE do not perform better than linear Pearson’s correlation, and in general, we find that complex methods are no more accurate than simple methods. The Z-SCORE method and Pearson correlation are the two best-performing unsupervised methods.

**Figure 8:**
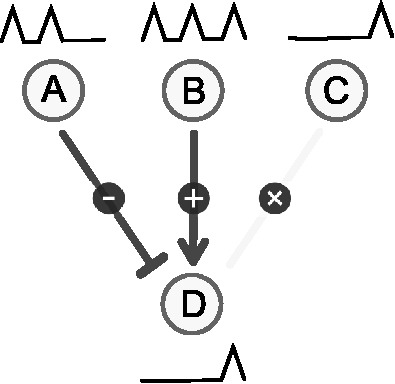
Gene A inhibits gene D, and gene B activates gene D. The resulting expression profile of gene D is, however, most similar to that of gene C, which does not regulate gene D.

A detailed analysis revealed that unsupervised approaches work well for simple network topologies (e.g. star topology) and networks with exclusively activating or inhibiting interactions, but fail for more complex cases (see Supplementary Material). Mixed regulatory interactions constitute a fundamental problem for unsupervised network inference as depicted in Figure 8.

Let gene A inhibit gene D but let gene B activate the same gene D. Given the expression profiles of genes A and B as shown in Figure 8, and assuming identical interaction weights but with opposite signs, the profile for gene D, resulting from a linear combination, is most similar to that of gene C and different from A or B. Consequently, the most appropriate but erroneous conclusion is to infer a regulatory relationship between C and D. Without any further information (e.g. knockouts, existing interactions) any method that infers interactions from the similarity of expression profiles alone is prone to fail in this common case. Schaffter *et al.* [12] identify other common network motifs and the methods that tend to infer them incorrectly, and Krishnan *et al.* [38] show that networks of a certain complexity cannot be reverse-engineered from expression data alone.

### Experimental data

We evaluated all inference methods on simulated and experimental data, and found the results to be in good agreement. Specifically, the supervised methods consistently achieved much higher accuracies than unsupervised methods on both simulated and experimental data, and prediction accuracies for simulated and experimental data were similar. Marbach *et al.* [8] report substantially lower accuracies (of unsupervised methods) for *S. cerevisiae* and attribute this to the increased regulatory complexity and prevalence of posttranscriptional regulation in eukaryotes and/or to the lower coverage of the *S. cerevisiae* network.

In our evaluations, the accuracies for *S. cerevisiae* were only slightly lower than for *E. coli*, but note that Marbach *et al.* [8] infer the complete network with >5000 nodes, while we randomly extract small subnetworks with 10–110 nodes and calculate an average accuracy. Any part of the *S. cerevisiae* network with low coverage would affect the average accuracy less than it would the overall accuracy. It may be unnecessary to invoke regulatory complexity, especially considering the high accuracies of the supervised methods.

Marbach *et al.* [8] furthermore suggest that incorporating additional information such as transcription-factor binding and chromatin modification data or promoter sequences might improve the accuracy of prediction. We have performed evaluations with added transcription-factor binding data but could not improve prediction accuracies (data not shown).

### Applications of inference methods

The inference of regulatory interactions from expression data has the potential to capture important associations between key genes regulating transcription in various biological conditions. Concerns about the accuracy of inference methods have led to the development of approaches that include GRN inference as one part of a broader computational framework. For example, He *et al.* [39] used modified correlation network inference to identify key genes involved in the immune suppressor function of human regulatory T cells. These authors combined two correlation-based approaches [36, 40] capable of identifying patterns of correlation across time-series microarray expression data. They then assessed the quality of their inferred networks by the extent to which interacting proteins shared biological process annotations, and selected key functional hubs from the inferred network using a metric computed from the results of literature mining and expression differences. They found that 6 of the top 10 hubs so ranked were already known to be involved in the suppressor function of regulatory T cells, and went on to experimentally characterize the novel role of the hub gene plasminogen activator urokinase (PLAU) in this suppressor function.

Similarly, Della Gatta *et al.* [41] applied the inference method ARACNE to reverse-engineer a genome-scale GRN in leukemia. To define the oncogenic regulatory circuits controlled by homeobox transcription factors TLX1 and TLX3, these investigators then used ChIP-chip data for these transcription factors along with expression data to identify direct targets of the TLX genes in the inferred network, requiring target genes to be differentially expressed between tumors that express TLX1 and TLX3. They then selected the most highly connected hub, RUNX1, as a putative master regulator of the TLX1 and 3 transcriptional programs, and validated the predictions of their network model with further ChIP-chip analysis of RUNX1.

These examples illustrate how GRN inference can productively be combined with downstream analysis: a GRN is initially inferred from experimental data, features of interest are identified based on network topology and interesting features are then ranked by applying additional criteria derived from experimental evidence, functional annotation or literature. Both He *et al.* [39] and Della Gatta *et al.* [41] used GRNs to identify highly connected nodes as features of interest, and then either rank these hubs, or refine the networks, by use of additional evidence. Interesting hubs prioritized in this way then form the basis of hypotheses that can be subjected to experimental investigation. Such hybrid approaches leverage the power of GRN inference methods, while controlling for variability in prediction performance by using additional criteria to select network features of interest.

## CONCLUSION

Perhaps the most important observation from this evaluation is the large variance in prediction accuracies across all methods. In agreement with Haynes and Brent [17], we find that a large number of repeats on networks of varying size is required for reliable estimates of the prediction accuracy of a method. Evaluations on single data sets—especially on real data—are unsuitable to establish differences in the prediction accuracy of inference methods.

On average, unsupervised methods achieve low prediction accuracies, with the notable exception of the Z-SCORE method, and are considerably outperformed by supervised and semi-supervised methods. Simple correlation methods such as Pearson correlation are as accurate as much more complex methods, yet much faster and parameterless. Unsupervised methods are appropriate for the inference only of simple networks that are entirely composed of inhibitory or activating interactions but not both.

The Z-SCORE method achieved the best prediction accuracy of all methods on knockout data. However, experimental knockouts cannot be performed systematically, or at all, in many important biological systems. The method also fails when a gene is regulated by an or-junction of two genes.

On multifactorial data, the supervised and semi-supervised methods achieved the highest accuracies; even with as few as 10% of known interactions, the semi-supervised methods still outperformed all unsupervised approaches. There was little difference in prediction accuracy for semi-supervised methods trained on positively labeled data only, compared with training on positive and negative samples. Apparently semi-supervised methods can effectively be trained on partial interaction data, and non-interaction data are not essential.

These results have important implications for the application of network inference methods in systems biology. Even the best methods are accurate only for small networks of relatively simple topology, which means that large-scale or genome-scale regulatory network inference from expression data alone is currently not feasible. If inference methods are to be applied to data of the scale generated by modern microarray platforms, a feature selection step is usually required to reduce the size of the inference problem; attempts to apply network inference to such large-scale datasets may be premature, and consideration should be given to focusing the biological question to use smaller-scale higher-quality experimental data.

Our analysis also indicates that certain kinds of biological data are more amenable for accurate network inference than others. Most microarray datasets are most similar to our multifactorial data sets, which yielded poorly inferred networks with unsupervised methods. Increasing the number of samples in the experiment (a common strategy to improve inference) does not in fact generate the hoped-for improvements. More useful are knockout data, which our simulations show contain more useful information, and support higher-quality inference. Biologists who wish to gain insight into regulatory architecture should consider these limitations when designing experiments.

To summarize, small networks (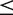
50 nodes) can be inferred with high accuracy (AUC 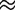 0.9) even with small numbers of samples using supervised techniques or the Z-SCORE method. However, even with the best-performing methods, large variations in prediction accuracy remain, and predictions may be limited to undirected networks without self-interactions.

### SUPPLEMENTARY DATA

Supplementary data are available online at http://bib.oxfordjournals.org/

**Key points**

- Prediction accuracies strongly depend on the complexity of the network topology.
- Supervised methods generally achieve higher prediction accuracies than unsupervised methods.
- Unsupervised inference methods are accurate only for networks with simple topologies.
- An exception is the unsupervised Z-SCORE method that shows the highest accuracy on simulated knockout data.
- Knockout data are more informative for network inference than knockdown or multifactorial data.

## FUNDING

Australian Research Council (DP110103384 and CE0348221).

## Supplementary Material

Supplementary Data
