# Supplementary material for: Supervised, semi-supervised and unsupervised inference of gene regulatory networks
Source: Brief Bioinform. 2013 May 21;15(2):195–211. doi: 10.1093/bib/bbt034 (PMC3956069; doi:10.1093/bib/bbt034)
Supplement: Supplementary Data [file supp_15_2_195__index.html]

Supervised, semi-supervised and unsupervised inference of gene regulatory networks — Supervised, semi-supervised and unsupervised inference of gene regulatory networks — Supplementary Data 

# Supervised, semi-supervised and unsupervised inference of gene regulatory networks

## Supplementary Data

files

**Files in this Data Supplement:**

- Supplementary Data - zip file
