# Supplementary material for: Supervised, semi-supervised and unsupervised inference of gene regulatory networks
Source: Brief Bioinform. 2013 May 21;15(2):195–211. doi: 10.1093/bib/bbt034 (PMC3956069; doi:10.1093/bib/bbt034)
Supplement: Supplementary Data [file supp_bbt034_suppl_data.zip › Supplementary.pdf]

Supplementary material

Supervised, semi-supervised and unsupervised inference of gene regulatory networks

Stefan R. Maetschke, Piyush B. Madhamshettiwar, Melissa J. Davis and Mark A. Ragan

April 11, 2013

1 Unsupervised

This sections contains additional data of unsupervised methods for different performance metrics and experimental data types.

1.1 Methods

The following three figures show the prediction performance of unsupervised methods for three different performance measures such as the Area Under the ROC curve (AUC), Matthew's Correlation Coefficient (MCC) and the F1-score. The threshold for the MCC and F1 score metrics were optimized. The AUC does not have a threshold that requires optimization.

All methods were evaluated on multi-factorial, knock-out, knock-down and averaged (all) data generated by GeneNetWeaver. Each evaluation was repeated 10 times over networks with 10,...,110 nodes, extracted from *E. coli* and *S. cerevisiae* networks.

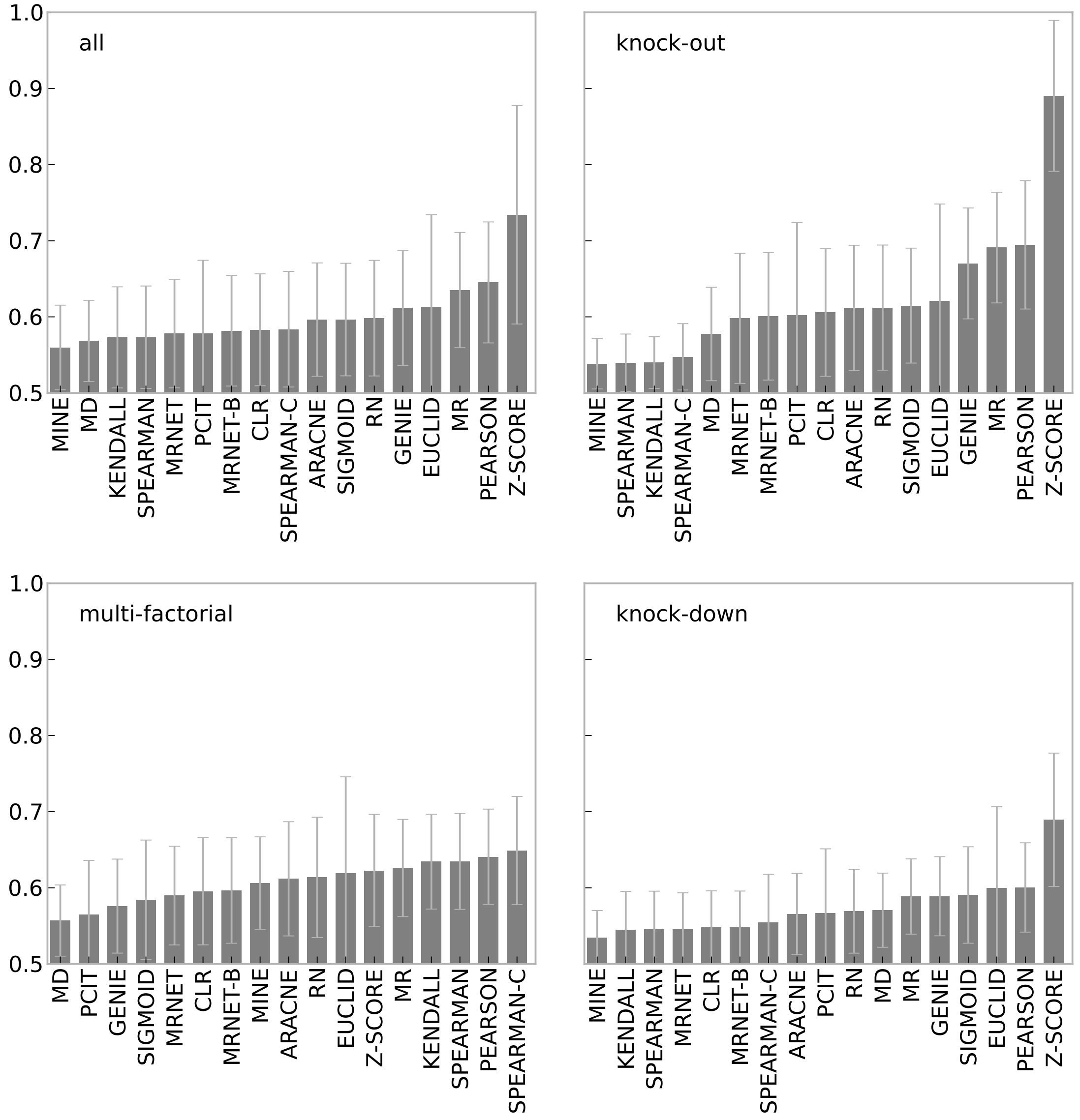

Figure 1: Prediction accuracy (AUC) of unsupervised methods for different experimental data types. Error bars show standard deviation.

While there are slight differences in the ranking of the methods depending on the chosen performance metric no dramatic shifts can be observed. Z-SCORE and PEARSON remain the best performing methods in all cases and the Z-SCORE method dominates all other methods for knock-out data.

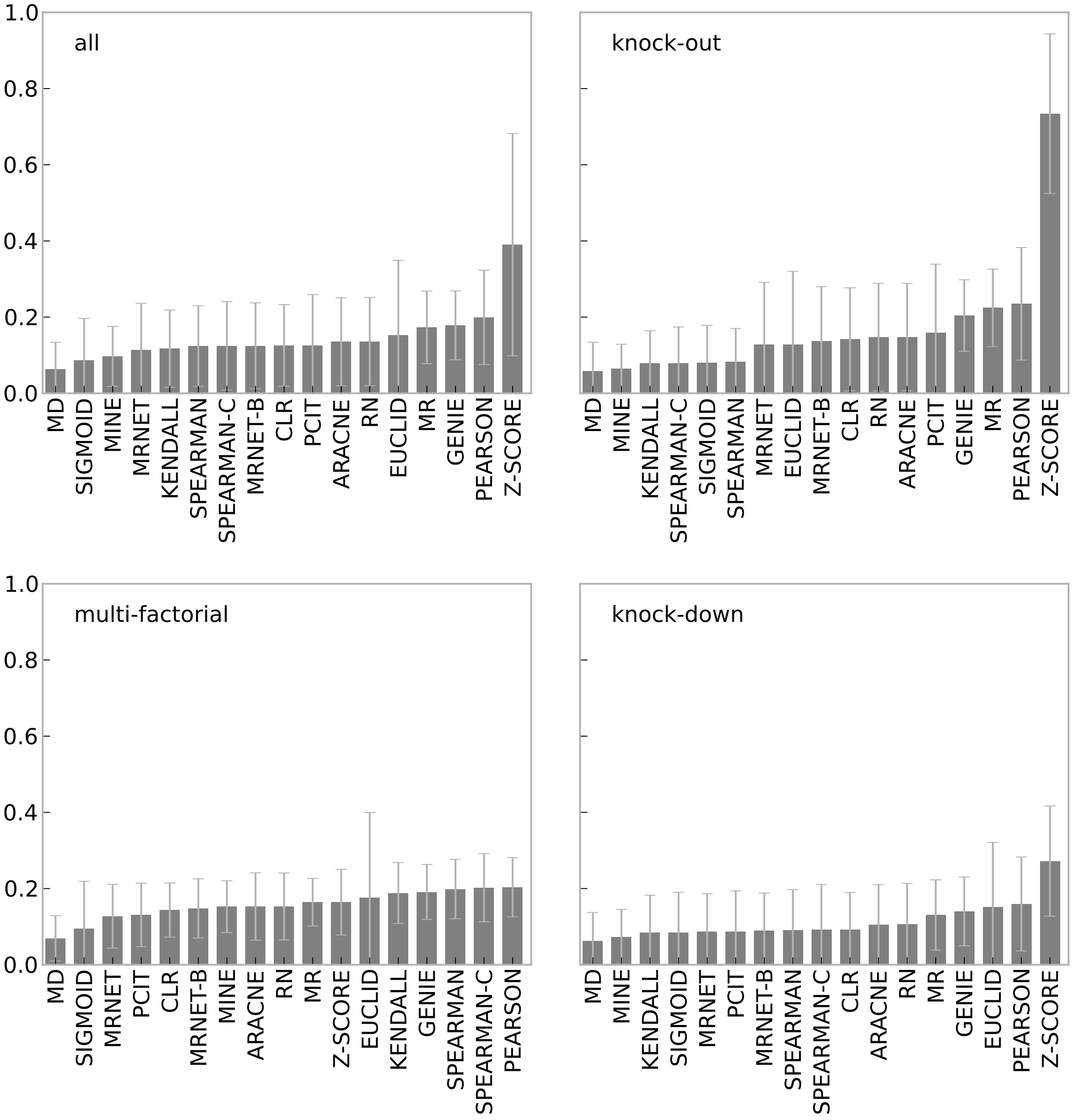

Figure 2: Prediction accuracy (MCC) of unsupervised methods for different experimental data types. Error bars show standard deviation.

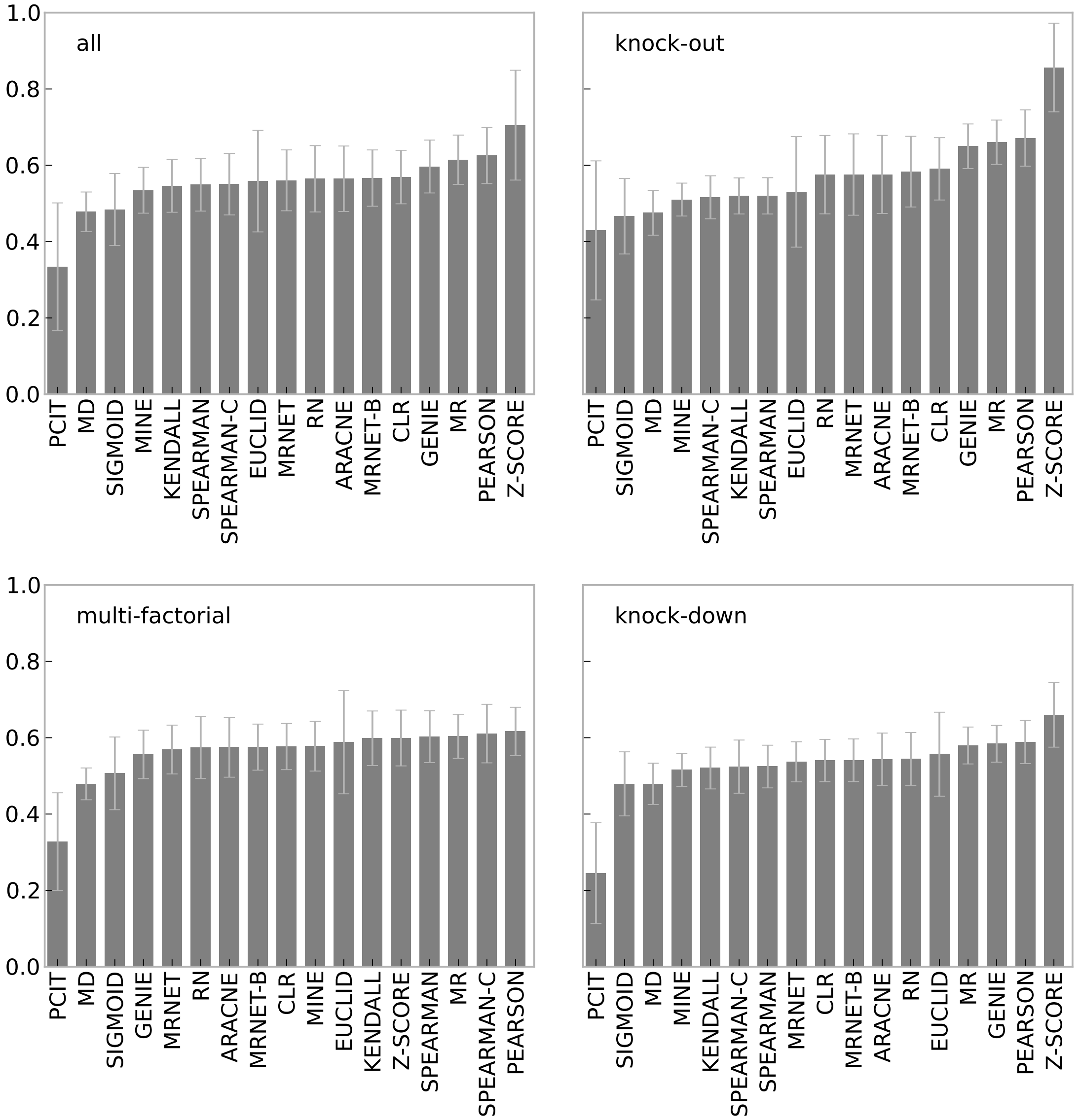

Figure 3: Prediction accuracy (F1-score) of unsupervised methods for different experimental data types. Error bars show standard deviation.

1.2 Network size

This section shows the prediction performance (AUC) of the unsupervised methods for networks with different node numbers and for the three experimental types (multi-factorial, knock-down, knock-out). All expression data were simulated with GeneNetWeaver and sub-networks were extracted from *E. coli*.

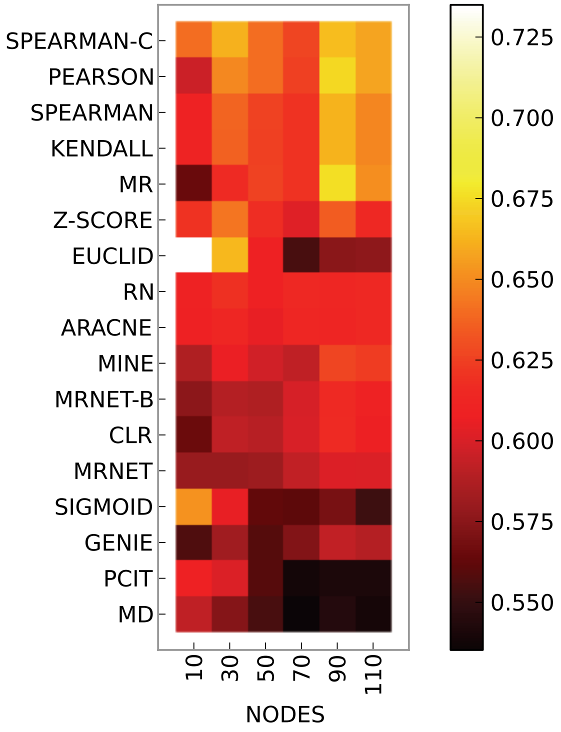

Figure 4: Prediction accuracy (AUC) of unsupervised methods on multi-factorial data for different network sizes.

Figure 4 reveals that the best performing unsupervised method on multi-factorial data is the EUCLID method but only on very small networks with 10 to 30 nodes. Correlation based methods such as PEARSON, SPEARMAN-C, SPEARMAN, KENDALL and some other methods show better performance on larger networks (90 and 110 nodes) than on smaller networks.

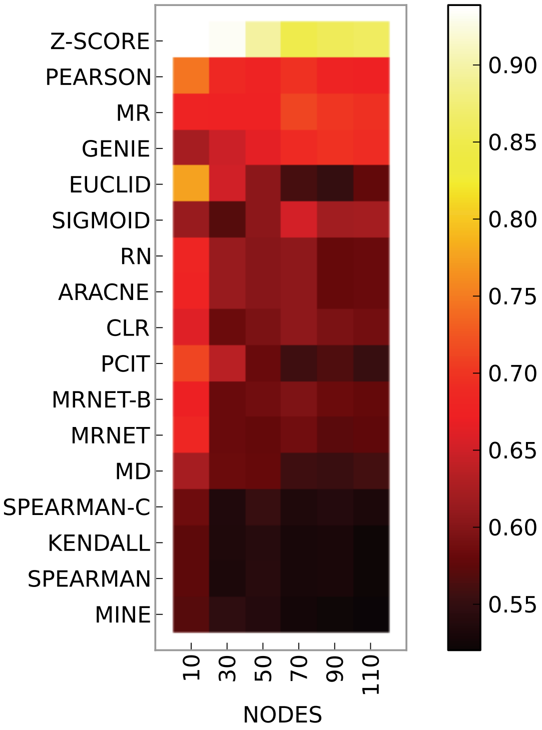

Figure 5: Prediction accuracy (AUC) of unsupervised methods on knock-out data for different network sizes.

On knock-out data the most accurate method is the Z-SCORE method. While the prediction accuracy of the Z-SCORE method decreases with network size it still clearly outperforms all other methods for networks of all sizes (see Figure 5). There is a general trend for most methods to perform better on the small 10-node network. Apart from PEARSON, all correlation based methods (SPEARMAN-C, SPEARMAN, KENDALL, MINE) achieve very low AUCs on knock-out data.

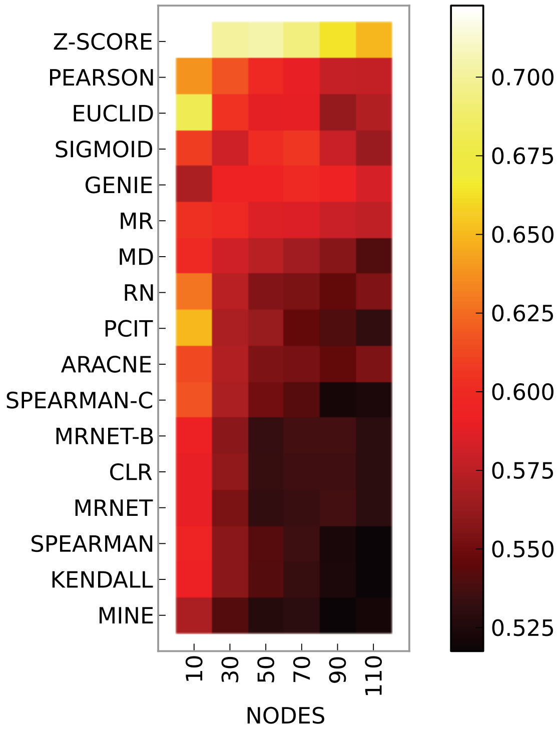

Figure 6: Prediction accuracy (AUC) of unsupervised methods on knock-down data for different network sizes.

The results on the knock-down data shown in Figure 6 are similar to the results on the knock-out data (see Figure 5). The Z-SCORE method remains the best performing method. The large majority of methods perform best on the small 10-node network – especially the EUCLID method, which was the best performer on networks of this size for multi-factorial data.

1.3 Network predictions

All evaluation showed large variations in the prediction accuracy of the methods. Even for very small networks with only 10 nodes the prediction accuracy can vary from perfect to completely wrong. To better understand the reasons causing the large variances we visualized the networks (out of 100) that were predicted with the highest and lowest accuracy, using Spearman's correlation as a network inference method and the AUC as performance metric. Sub-networks with 10 nodes were extracted from the *E. coli* network and expression data were simulated with GeneNetWeaver.

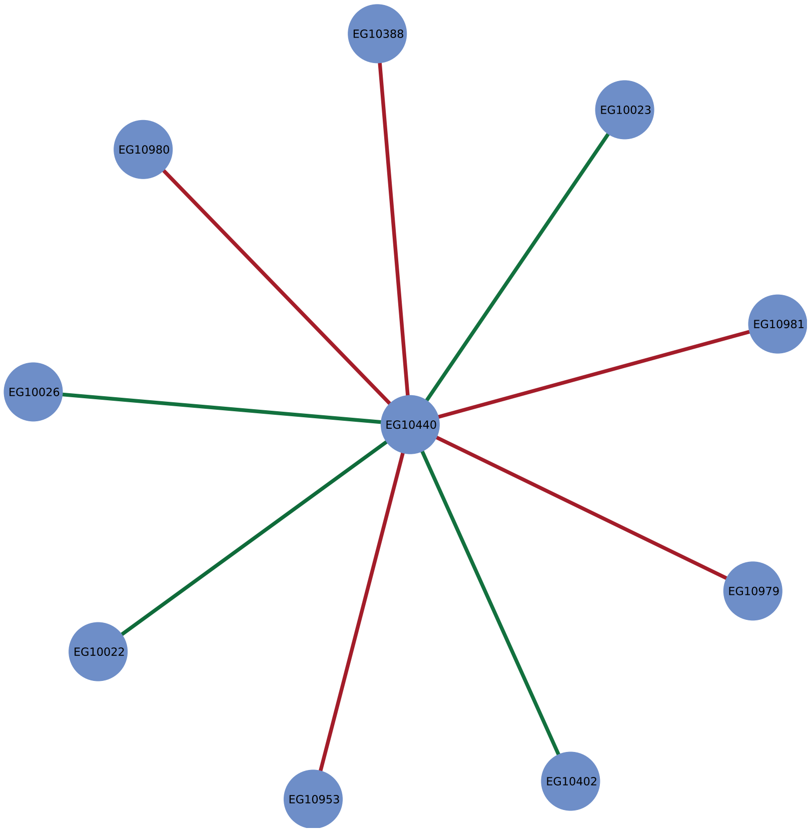

Figure 7: True network where Spearman's correlation failed to recover the topology (AUC = 0.508). Green means activating, and red means inhibiting interactions

Figure 7 shows a true network where Spearman's correlation failed to recover the topology (AUC = 0.508). Note that some interactions are activating (green) and some interactions are inhibiting (red), which results in a more complex dynamic of the network than a network with exclusively activating or inhibiting interactions. In contrast, Figure 8 shows the true network where Spearman's correlation inferred the network topology close to perfect (AUC = 0.971).

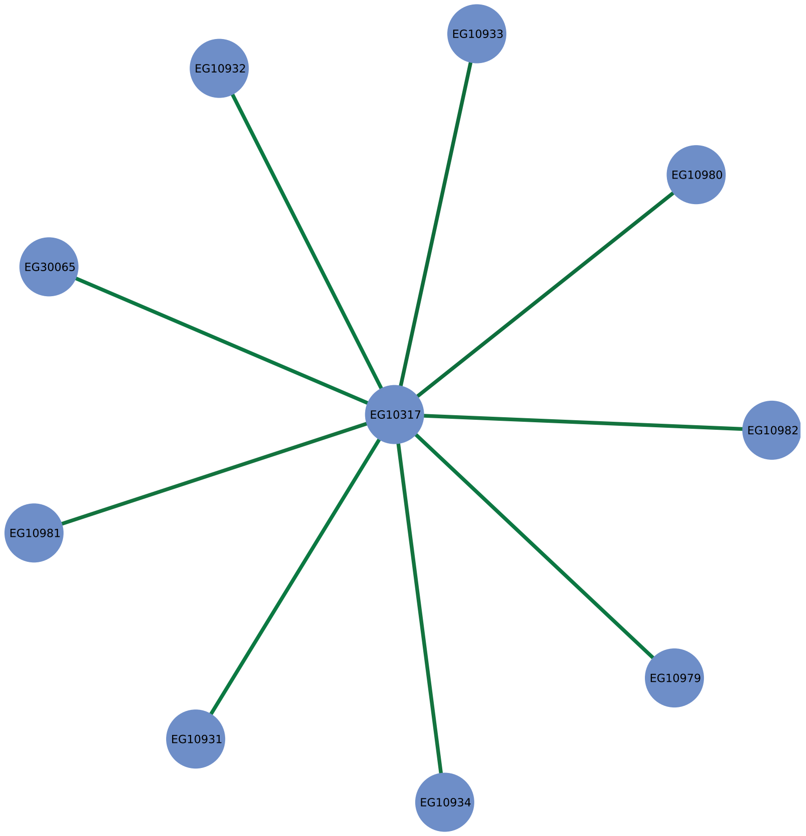

Figure 8: True network where Spearman's correlation recovered the topology accurately (AUC = 0.971). The network has only activating (green) interactions.

In general, networks with exclusively activating or inhibiting interactions and simple topologies (e.g. star topology) can be inferred accurately with unsupervised methods, even on multi-factorial data. However, networks with complex topologies or a mix of activating and inhibiting interactions typically cannot be recovered reliably from multi-factorial data.

2 Supervised

This section compares supervised, semi-supervised and unsupervised methods, using three different performance metrics such as the Area Under the ROC curve (AUC), Matthew's Correlation Coefficient (MCC) and the F1-score.

All methods were evaluated on multi-factorial, knock-out, knock-down and averaged (all) data generated by GeneNetWeaver. 5-fold cross-validation was used and each evaluation was repeated 10 times over networks with 30 nodes, extracted from *E. coli*.

The results show little difference in the ranking of the methods for different performance metrics. The Z-SCORE method achieves the highest accuracies on the knock-out data but performs worst on multi-factorial data. SPEARMAN typically shows the lowest prediction accuracy and semi-supervised methods are effectively ranked according to the percentage of labeled data used. No distinction between semi-supervised methods trained on positives and negatives and methods trained on positives-only can be observed.

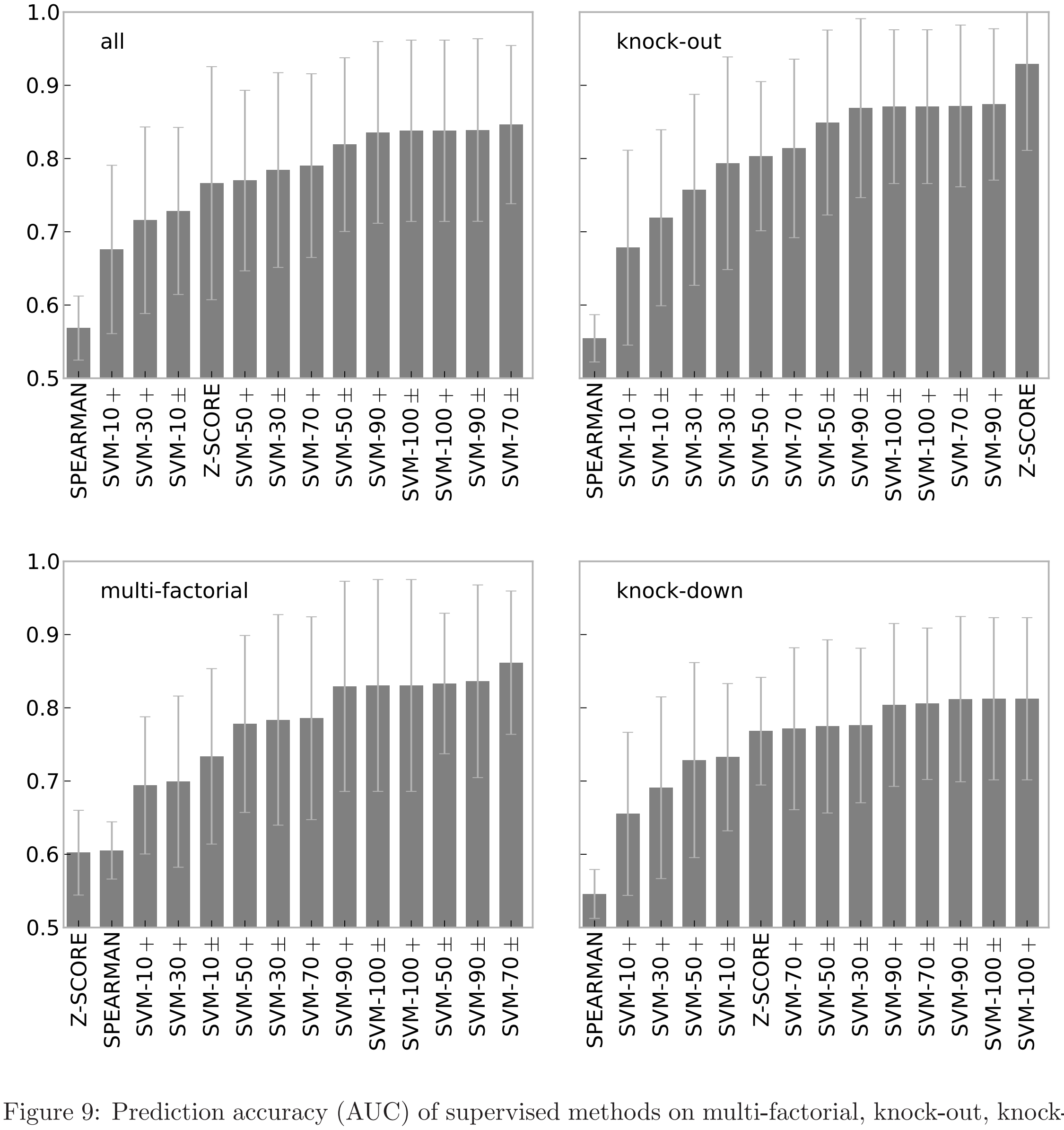

Figure 9: Prediction accuracy (AUC) of supervised methods on multi-factorial, knock-out, knock-down and averaged (all) data generated by GeneNetWeaver. Error bars show standard deviation.

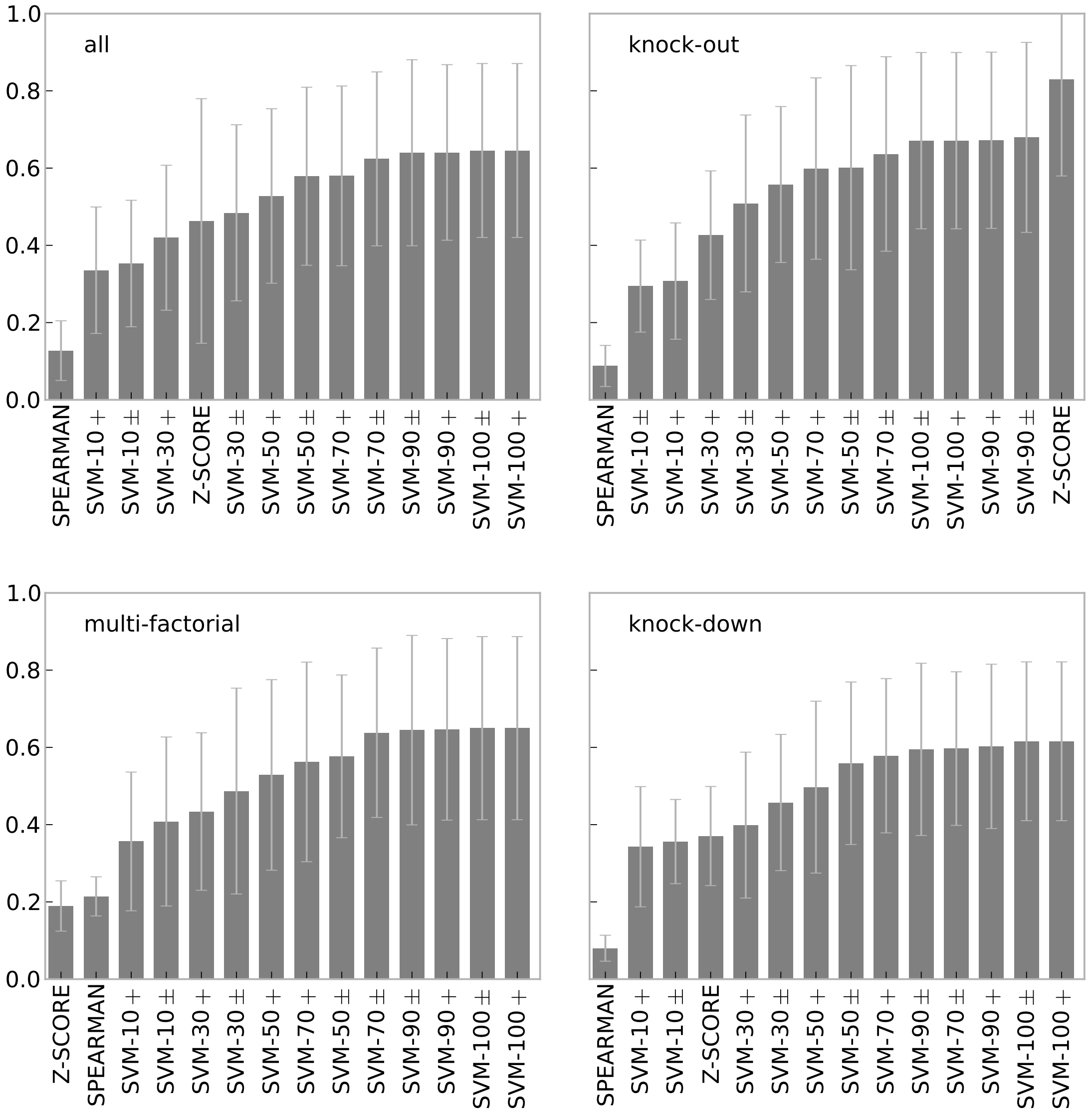

Figure 10: Prediction accuracy (MCC) of supervised methods on multi-factorial, knock-out, knock-down and averaged (all) data generated by GeneNetWeaver. Error bars show standard deviation.

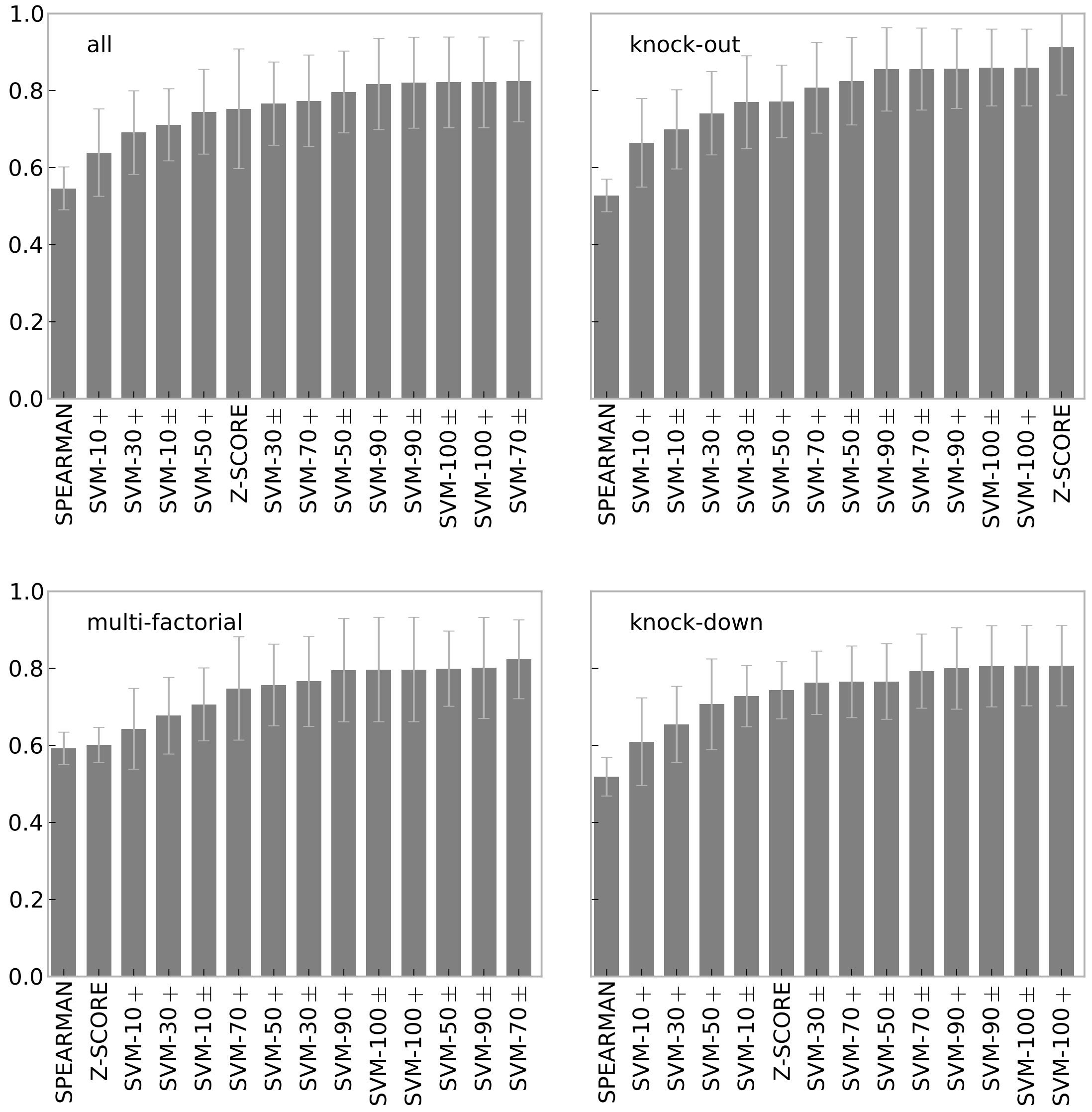

Figure 11: Prediction accuracy (F1-score) of supervised methods on multi-factorial, knock-out, knock-down and averaged (all) data generated by GeneNetWeaver. Error bars show standard deviation.

3 Experimental

We compared the prediction accuracy of unsupervised methods on experimental data for *E. coli* and *S. cerevisiae* when using all samples of the expression data set or only the first 30 samples. The results show no increase in accuracy for the best performing methods when using all data but most other methods benefit from the additional data. To summarize, additional expression data tend to equalize the prediction accuracy of the methods but do not improve on the peak performance.

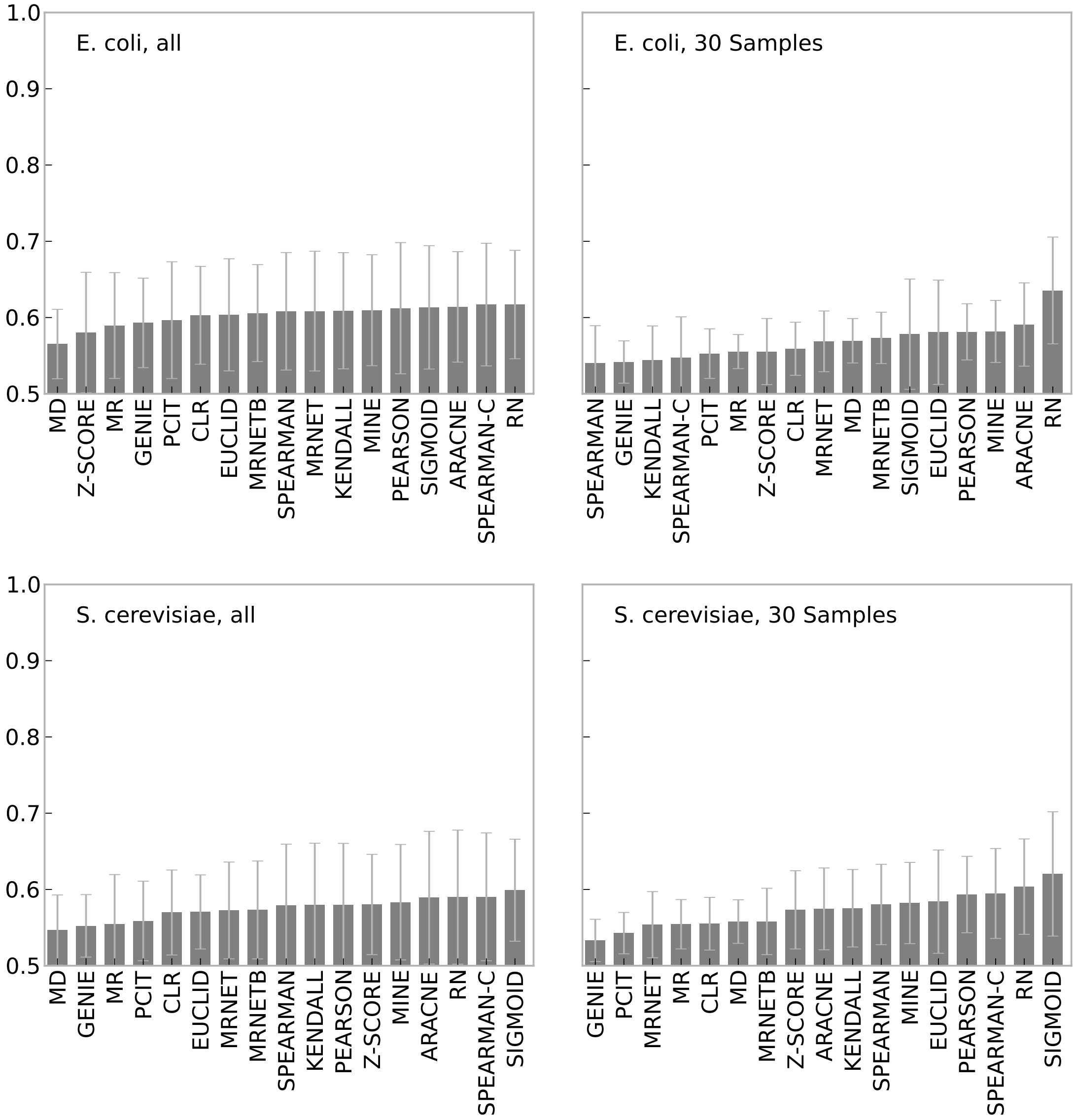

Figure 12: Prediction accuracy (AUC) of unsupervised methods on the experimental data using all data or only 30 samples. Error bars show standard deviation.
